# RIPK3 is spatially associated with cartilage degeneration in osteoarthritis: integrative transcriptomic and histological analysis

**DOI:** 10.1186/s13018-025-06321-x

**Published:** 2025-11-10

**Authors:** Lei Wang, Jing Tang, Lei Niu, Zihua Li, Lang Wu, Wei Zhao, Guanghui Wang

**Affiliations:** 1 Department of Trauma and Joint Surgery, The Fifth People’s Hospital of Ningxia Hui Autonomous Region, Shizuishan, 753000 Ningxia China; 2 School of Basic Medicine, Ningxia Medical University, Yinchuan, 750004 China; 3 Ningxia Key Laboratory of Prevention and Control of Common Infectious Disease, Ningxia Medical University, Yinchuan, 750004 China; 4 Department of Stomatology, The People’s Hospital of Ningxia Hui Autonomous Region, Yinchuan, 750000 China; 5 Department of Hepatobiliary Surgery, The People’s Hospital of Ningxia Hui Autonomous Region, Yinchuan, 750000 China

**Keywords:** RIPK3, Osteoarthritis, Cartilage degeneration, Multi-regional transcriptomics

## Abstract

**Background:**

Osteoarthritis (OA) is a degenerative joint disease characterized by progressive cartilage loss, yet its molecular underpinnings remain incompletely defined. This study aimed to investigate the regional expression pattern of receptor-interacting protein kinase 3 (*RIPK3*) in OA cartilage and its association with histopathological severity.

**Methods:**

Cartilage samples were collected from OA patients and categorized into normal tissue (NT), junction tissue (JT), and lesioned tissue (LT). RNA sequencing was performed to identify differentially expressed genes (DEGs) between NT and JT. Gene enrichment analysis was conducted to explore functional pathways. Immunofluorescence staining was used to validate RIPK3 protein expression and localization.

**Results:**

Transcriptomic analysis identified 140 differentially expressed genes (DEGs) between NT and JT, with several genes, including *RIPK3*, *CCL19*, and *ITLN1*, significantly up-regulated in JT. *RIPK3* expression showed a log_2_ Fold Change of 2.17 (*p* < 0.01) and displayed higher protein abundance in LT (3.66-fold vs NT), concordant with the transcriptomic trend. This increase correlated with more severe histopathological damage (Mankin score: LT 33.0 vs. NT 5.6; OARSI score: LT 15.8 vs. NT 1.6). Functional enrichment analysis associated RIPK3 with necroptosis, immune signaling and extracellular matrix catabolism. Immunofluorescence staining confirmed spatial accumulation of RIPK3 in LT, consistent with transcriptomic data.

**Conclusions:**

RIPK3 is spatially enriched in degenerative regions of OA cartilage and associates with histopathological damage, suggesting its potential involvement in disease progression. These findings provide new insight into the molecular landscape of OA and support RIPK3 for further evaluation as a biomarker and potential therapeutic target.

## Introduction

**Osteoarthritis (OA)** is one of the most common degenerative joint diseases, affecting over 300 million individuals globally, with significant implications for both public health and the patients' quality of life [1]. OA primarily affects weight-bearing joints such as the knees and hips, where repetitive mechanical stress, exacerbated by aging and metabolic dysregulation, leads to progressive joint tissue damage [2, 3]. The ongoing demographic shift toward aging populations, along with the rising prevalence of obesity, has further exacerbated the global burden of OA, positioning it as a leading cause of musculoskeletal disability [4]. Current therapeutic strategies for OA are largely symptomatic, focusing on pain relief and functional preservation. Common treatments include analgesics, nonsteroidal anti-inflammatory drugs (NSAIDs), and intra-articular injections of corticosteroids or hyaluronic acid, which provide only temporary relief and do not address the underlying disease processes [5, 6]. For patients with end-stage OA, total joint replacement (TJR) is the most effective intervention; however, its applicability is often limited by the invasiveness of the procedure and the presence of comorbidities, especially in elderly patients [6]. These limitations underscore the urgent need to identify the molecular drivers of OA pathogenesis, which could pave the way for the development of disease-modifying therapies.

OA pathogenesis is multifactorial, involving a range of tissue-level changes. Key pathological features include articular cartilage degradation, subchondral bone sclerosis, osteophyte formation, and synovitis [7]. The primary pathological event in OA is the irreversible loss of articular cartilage, a tissue that is avascular and aneural, thus exhibiting limited intrinsic repair capacity [8]. Chondrocytes within the cartilage exhibit disrupted homeostasis, primarily due to an imbalance between catabolic and anabolic processes, leading to extracellular matrix degradation and progressive loss of tissue integrity [5, 9]. Additionally, subchondral bone remodeling alters the mechanical properties of the joint, contributing to further cartilage damage and pain through nociceptive signaling [10]. Longitudinal μCT and histologic analyses in DMM mice demonstrate that RANKL-mediated osteoclastic loss of subchondral bone volume and mineral density occurs at a very early stage and precedes histological cartilage degeneration, with high-turnover bone metabolism further exacerbating these changes and culminating in uncoupled remodeling [11].

Recent studies suggest that receptor-interacting protein kinase 3 (*RIPK3*), a key regulator of programmed cell death, particularly necroptosis, may play a critical role in OA progression. Necroptosis is an inflammation-related form of programmed cell death, which could be involved in inflammatory amplification, chondrocyte death, and cartilage degeneration in OA [12]. In addition to necroptotic signaling, autophagy–PI3K/Akt/mTOR pathways critically regulate cartilage homeostasis. Blocking *miR-103-3p* reinstates CPEB3, dampens PI3K/Akt/mTOR signaling, boosts autophagy, and reduces apoptosis and MMP/ADAMTS-mediated matrix degradation in chondrocytes [13]. Although elevated *RIPK3* expression has been associated with the severity of cartilage damage, the specific regional expression pattern of *RIPK3* within OA cartilage and its precise relationship to disease progression remain unclear. Thus, the exact role and expression dynamics of *RIPK3* in OA progression warrant further investigation. In the present study, we performed high-throughput RNA sequencing on cartilage samples collected from OA patients undergoing TJR. These samples were obtained from different anatomical regions, including normal tissue (NT), junction tissue (JT), and lesioned tissue (LT), to identify region-specific molecular changes associated with disease severity. This multi-regional approach enabled the identification of differentially expressed genes (DEGs) associated with cartilage degeneration. Among these DEGs, *RIPK3* emerged as a key molecule whose expression levels potentially correlate with the degree of tissue damage. Immunofluorescence staining was further conducted to validate the protein expression of *RIPK3*, supporting its potential involvement in OA progression. This study aims to clarify the potential role of *RIPK3* as a molecular marker for cartilage degeneration, thus providing new directions for mechanistic research and therapeutic strategies in OA.

## Materials and methods

### Clinical specimen collection and processing

This study was conducted in accordance with the ethical principles of the Declaration of Helsinki. Written informed consent was obtained from all participants prior to enrollment. Cartilage tissues were collected intraoperatively from six patients (four females, two males; mean age: 68.83 ± 3.19 years) diagnosed with primary osteoarthritis (OA) and undergoing TJR surgery. Based on pathological features, cartilage tissues were categorized into three groups: LT (regions with severe degeneration, e.g., cartilage defects or fibrosis); JT (junction zones between lesioned and normal cartilage); NT (macroscopically intact cartilage without degenerative changes). Tissue samples (1 cm^3^ per region) were snap-frozen in liquid nitrogen and stored at – 80 °C.

### RNA extraction and library preparation

Total RNA was extracted from cartilage tissues using TRIzol reagent (Invitrogen, USA). RNA integrity was evaluated using an Agilent Bioanalyzer 2100 system with an RNA 6000 Nano LabChip kit (Agilent, USA), and samples with RNA integrity number (RIN) ≥ 7 were considered qualified. Due to severe RNA degradation in lesion areas (RIN < 5), only transitional and preserved tissues from six patients (12 samples total) were selected for sequencing. Libraries were prepared following the Illumina TruSeq Stranded mRNA protocol. First, mRNA enrichment was performed by purifying polyadenylated RNA from 5 μg of total RNA using Dynabeads Oligo(dT) (Thermo Fisher, USA). Next, during fragmentation and synthesis, the mRNA was fragmented into 200–300 bp fragments in a divalent cation buffer at 94 °C, followed by double-stranded cDNA synthesis using SuperScript™ II Reverse Transcriptase (Invitrogen, USA). Finally, in the library construction step, end repair, A-tailing, adapter ligation, and PCR amplification (15 cycles) were carried out. The resulting libraries, with an insert size of 300 ± 50 bp, were quality-checked using an Agilent 2100 system and sequenced on an Illumina NovaSeq 6000 platform with 150 bp paired-end reads.

### Transcriptomic data analysis and gene interaction network construction

#### Data preprocessing and alignment

Raw sequencing reads were filtered using Fastp (v0.23.1) to remove adapter-contaminated reads, polyA/G-rich reads (> 10%), reads containing > 5% unknown bases (N), or low-quality reads (> 20% bases with Q ≤ 20). Clean reads were assessed using FastQC (v0.11.9) for Q20/Q30 scores, GC content, and read length distribution. HISAT2 (v2.2.1) was used to align reads to the human reference genome GRCh38 (Ensembl release 104) with parameters “-dta-rna-strandness RF” for strand-specific alignment.

#### Gene expression quantification and differential analysis

Transcript assembly and expression quantification (FPKM values) were performed using StringTie (v2.1.7). Differential expression analysis between groups was conducted using DESeq2 (v1.38.3), which applies a negative binomial model. DEGs were defined as those with |log_2_FoldChange|≥ 1 and Benjamini-Hochberg-adjusted FDR < 0.05. DEG expression patterns were visualized via volcano plots (ggplot2, v3.4.0) and heatmaps (pheatmap, v1.0.12; Euclidean distance, Ward.D2 clustering).

#### Functional enrichment and pathway analysis

Significant DEGs were functionally annotated as follows: Gene Ontology (GO): Enriched terms (Biological Process, Cellular Component, Molecular Function) were identified using ClusterProfiler (v4.6.2) with the 2023 GO database (FDR < 0.05), and results were displayed as bubble plots (Rich Factor vs. gene counts); KEGG Pathways: Enriched pathways were analyzed using the KEGG Orthology database (2023-Q2) and visualized as bar plots (top 20 pathways, FDR < 0.05).

#### Hub gene interaction network

Ten hub genes from enriched KEGG pathways were input into GENEMANIA (https://genemania.org/) to construct an interaction network (species: Homo sapiens). Network parameters included co-expression, physical interactions, genetic interactions, and pathway co-localization data, with edge weights optimized automatically. Cytoscape (v3.9.1) was used for topological analysis, calculating degree centrality and betweenness centrality to identify key regulatory genes.

### Histological and immunofluorescence staining

#### Paraffin section preparation

Tissues were fixed in 4% paraformaldehyde for 24 h, decalcified in 25% EDTA for 2 weeks with daily solution replacement, dehydrated in graded ethanol, embedded in paraffin (Leica EG1150H), and sectioned into 4 μm slices.

#### H&E staining and histopathological evaluation

Histological sections underwent xylene dewaxing, graded ethanol rehydration, and were stained with hematoxylin (5 min) and eosin (1 min). Differentiation was performed using hydrochloric acid–ethanol (30 s), followed by aqueous rinsing and neutral resin mounting. A panoramic scan of the slices was conducted using CaseViewer software. Cartilage degeneration was evaluated using the modified Mankin scoring system [14] and the Osteoarthritis Research Society International (OARSI) histopathology grading system [15]. The scoring criteria are detailed in Table 1 The scoring workflow comprised three phases: (1) single-field evaluation, with independent scoring at 10 × , 20 × , and 40 × magnifications; (2) total score calculation by summing field-specific Mankin (0–42) and OARSI (0–18) values; and (3) regional stratification, where NT, JT, and LT scores were analyzed as mean ± SD.

**Table 1:** Histopathological scoring criteria for OA cartilage degeneration

Scoring system	Evaluation domains	Scoring criteria	Score range (per field)	Total score (3 fields)
Mankin system	Structural integrity	0 (intact) – 6 (severe damage)	0–14	0–42
	Cellular Morphology	0 (normal) – 3 (abnormal)		
	Tidemark continuity	0 (continuous) – 1 (disrupted)		
	Matrix staining homogeneity	0 (uniform) – 4 (uneven loss)		
OARSI system	Depth of degeneration	0 (intact) – 6 (full-thickness collapse)	0–6	0–18
	Horizontal spread	Qualitative assessment		

#### Immunofluorescence staining

Antigen retrieval was performed using EDTA buffer (pH 8.0) with microwave heating. Sections were blocked with 3% BSA for 30 min. Sections were incubated overnight at 4 °C with rabbit primary antibodies (1:200), including: Anti-XCL1 + XCL2 antibody (Abcam, UK); Rabbit Anti-RIPK3 antibody (Bioss, China); SIRPG antibody (Affinity, USA); IL-12RB1 polyclonal antibody (Sangon Biotech, China); CCL19 polyclonal antibody (Sangon Biotech, China); ITLN1/2 polyclonal antibody (Sangon Biotech, China). After primary antibody incubation, sections were treated with CY3-conjugated secondary antibodies (1:500, 50 min, room temperature), counterstained with DAPI (10 min, dark), and mounted with anti-fade medium. Images were acquired, and a panoramic scan of the slices was conducted using CaseViewer software (excitation: DAPI 358 nm, CY3 552 nm). Semi-quantitative analysis was performed using ImageJ v1.53 k. Three non-overlapping fields of view were randomly captured per specimen. Following CY3 channel separation, Gaussian filtering (σ = 2 pixels) was applied for noise reduction. Positive signal regions were segmented using Otsu’s automatic thresholding algorithm. Integrated fluorescence density and regional area (μm^2^) were measured, and relative fluorescence intensity per unit area was calculated as MFI = Integrated Density / Area.

### Statistical analysis

Data were analyzed using R software (v4.2.0) and GraphPad Prism (v9.3.1). Continuous variables were expressed as mean ± standard deviation (SD). The normality of data distribution was assessed using the Shapiro–Wilk test. Comparisons between two groups were performed using the Student’s t-test or Mann–Whitney U test for normally distributed or non-normally distributed data, respectively. For comparisons among more than two groups, one-way analysis of variance (ANOVA) followed by Tukey’s post-hoc test was used. Chi-square tests were used for categorical data. A *p*-value < 0.05 was considered statistically significant. For DEGs analysis, a Benjamini–Hochberg adjusted p-value (FDR) < 0.05 and | log_2_ fold change |≥ 1 were considered as significant thresholds. Due to RNA degradation in the LT samples, sequencing was performed only on the JT samples and the NT samples (n = 6). However, in the subsequent pathological analysis, n = 5 per group due to severe tissue degradation in some samples. The pathological groups were classified as Normal, Junction, and Lesion groups. Differences between these groups were evaluated using the appropriate statistical tests, and results were presented based on the available sample size at each stage of analysis.

## Results

### Histopathological characteristics of cartilage degeneration in osteoarthritic

**Fig. 1:**
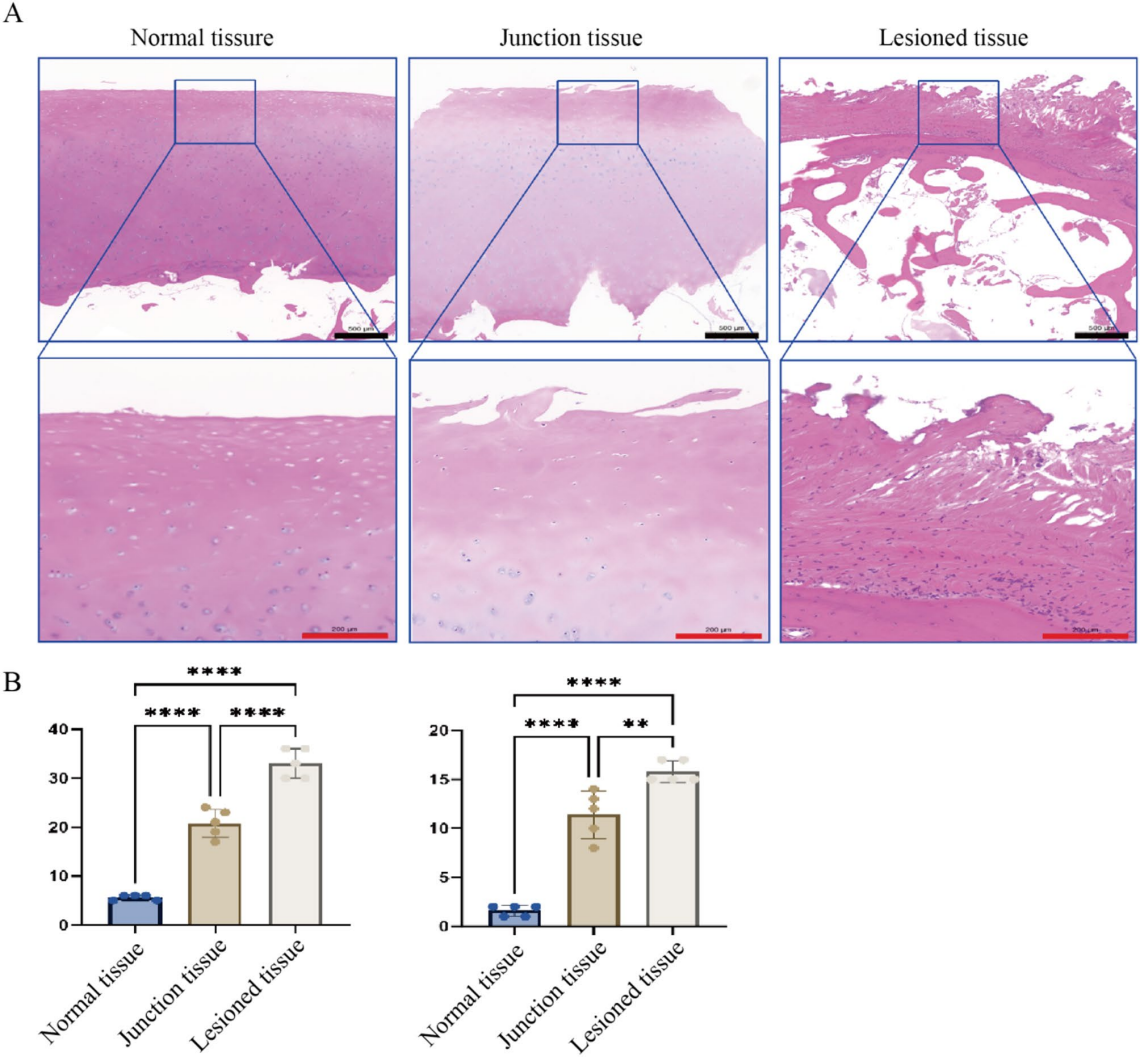
Histopathological characteristics of osteoarthritic cartilage degeneration. **A** Representative H&E-stained images of different cartilage regions, Scale bars:black-500 μm (upper panels); red-100 μm (lower panels). **B** Mankin and OARSI scores for the three regions, showing significant differences. Data are presented as mean ± SD. Statistical significance: *p* < 0.01 (**), *p* < 0.0001 (****), one-way ANOVA

Histomorphological analysis using hematoxylin and eosin (H&E) staining, combined with evaluations using the Mankin and OARSI scoring systems, demonstrated significant regional pathological gradients in osteoarthritic cartilage degeneration (Fig. 1A). The NT samples retained a characteristic hyaline cartilage structure, with chondrocytes arranged in single or columnar layers, a uniform nuclear-to-cytoplasmic ratio, and homogeneous basophilic staining of the extracellular matrix. Type II collagen fibers exhibited continuous distribution at the calcified-noncalcified cartilage interface. The JT samples displayed early degenerative features, including superficial microcracks, focal disruptions of the tidemark, clustered chondrocyte proliferation, and reduced basophilic staining intensity in the matrix. The LT samples exhibited end-stage degeneration, characterized by full-thickness cartilage loss, increased subchondral trabecular bone, and elevated marrow fibrosis. Quantitative analysis revealed significant differences in pathological scores across the three regions. The NT samples had a Mankin score of 5.6 ± 0.55 and an OARSI score of 1.6 ± 0.55, consistent with intact cartilage architecture. Degeneration was significantly worse in the JT samples, with Mankin and OARSI scores rising to 20.8 ± 2.86 and 11.4 ± 2.41, respectively (*p* < 0.001). The LT samples exhibited severe degeneration, reflected by further increases in Mankin (33.0 ± 3.0) and OARSI (15.8 ± 1.10) scores (*p* < 0.001). One-way ANOVA confirmed statistically significant differences among the three regions for both scoring systems (*p* < 0.001).

These histopathological assessments confirmed a clear gradient in cartilage degeneration severity across the anatomical regions sampled, providing a reliable pathological basis for subsequent molecular expression analyses.

### Transcriptomic changes in osteoarthritic cartilage

**Fig. 2:**
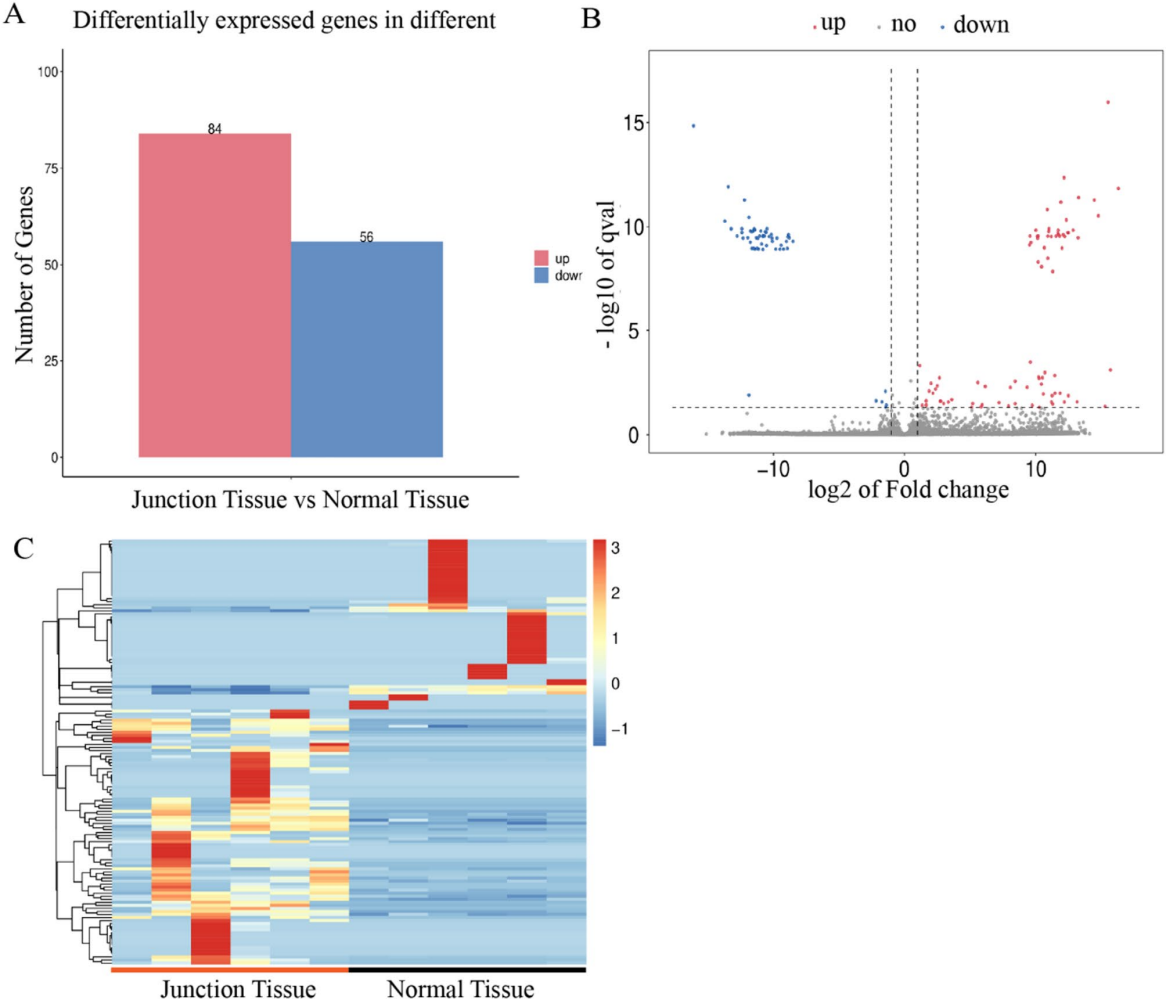
Transcriptomic alterations between JT and NT cartilage in osteoarthritis. **A** Bar plot showing the number of significantly up-regulated (n = 84) and down-regulated (n = 56) DEGs identified between JT and NT samples (FDR < 0.05, |log_2_FC|≥ 2). **B** Volcano plot highlighting significantly up-regulated (red) and down-regulated (blue) genes; dashed lines represent the thresholds for statistical significance. **C** Hierarchical clustering heatmap showing distinct gene expression profiles between JT and NT samples. Warmer colors indicate higher expression levels, and cooler colors indicate lower expression levels

To characterize the region-specific transcriptomic alterations in osteoarthritic cartilage, a differential gene expression analysis was conducted between NT and JT samples using stringent criteria (FDR < 0.05, |log_2_FC|≥ 2). A total of 140 DEGs were identified, including 84 up-regulated and 56 down-regulated transcripts (Fig. 2A). As visualized in the volcano plot (Fig. 2B), several DEGs exhibited exceptionally high fold changes (|log_2_FC|> 10), such as *ITLN1* (log_2_FC = 14.45), *CCL19* (log_2_FC = 12.84), *XCL1* (log_2_FC = 12.18), *SIRPG* (log_2_FC = 10.94), and *IL21R* (log_2_FC = 10.72). Conversely, genes such as *BLOC1S5-TXNDC5* (log_2_FC =  − 16.09) and *SCGB3A2* (log_2_FC =  − 12.40) were markedly down-regulated. Hierarchical clustering analysis (Fig. 2C) revealed a distinct segregation between NT and JT samples, reflecting robust intra-group consistency and region-specific gene expression profiles.

Of particular interest, *RIPK3*—although not among the top DEGs in terms of fold change—was significantly upregulated in the JT region (log_2_FC = 2.17, *p* < 0.01), suggesting a potential regulatory role in OA-related tissue remodeling. To further elucidate the biological relevance of the identified DEGs, they were classified into three major functional categories: (1) inflammation-related genes (e.g., *CCL19*, *XCL1*, *SIRPG*), (2) extracellular matrix-associated genes (e.g., *ITLN1*), and (3) immune regulatory genes (e.g., *IL21R*, *SIRPG*), all of which showed significant differential expression in JT samples. Table 2 presents a subset of representative genes with the most statistically significant expression differences (*p* < 0.001), highlighting molecular signatures that may underlie early OA pathogenesis.

**Table 2:** Log_2_FC and *p* values of some significantly DEGs

Up-regulate gene	Log2 FC	*P value*	Down-regulate gene	Log2 FC	*P value*
*ITLN1*	14.45	1.04E-15	*BLOC1S5-TXNDC5*	− 16.09	7.23E-20
*CCL19*	12.84	8.48E-14	*SCGB3A2*	− 12.40	5.64E-14
*XCL1*	12.18	8.68E-05	*TRAV8-6*	− 12.32	5.25E-13
*SIRPG*	11.85	2.86E-05	*POC1B-GALNT4*	− 11.87	1.07E-14
*IL21R*	2.99	1.05E-04	*CEMP1*	− 11.66	1.25E-13
*MMP25*	2.27	6.32E-04	*PRR9*	− 11.31	4.68E-13
*RIPK3*	2.17	2.86E-05	*MPPED2*	− 1.47	2.24E-05
*DGKI*	2.03	8.64E-06			
*ADAMTS2*	1.88	2.17E-05			

Collectively, these transcriptomic findings underscore the presence of pronounced inflammatory signaling and extracellular matrix imbalance in the JT region. *RIPK3*, in conjunction with other critical DEGs, may constitute part of a molecular network contributing to OA-associated cartilage degeneration. To further delineate the downstream mechanisms and pathways involved, functional enrichment analysis and gene interaction network modeling were performed.

### Functional enrichment and interaction network analysis of key genes

**Fig. 3:**
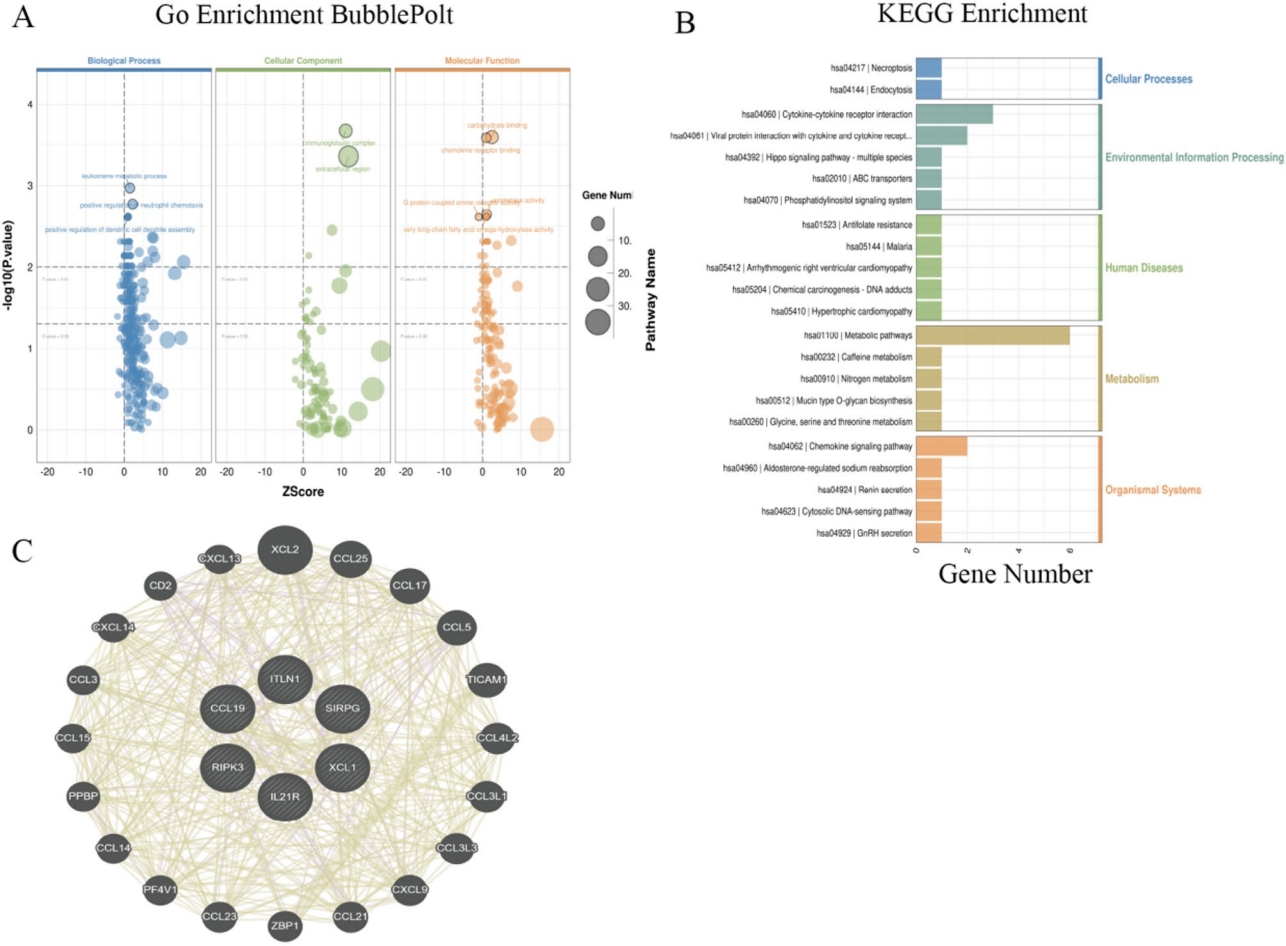
Functional enrichment analysis and gene interaction network of key DEGs. **A** GO enrichment analysis of DEGs, categorized into Biological Process, Cellular Component, and Molecular Function. Bubble size represents gene count, with –log_10_(*p*-value) on the y-axis and Z-score on the x-axis. **B** KEGG pathway analysis of DEGs, with pathways grouped into major functional categories. Gene count is plotted on the x-axis. **C** Gene interaction network constructed using GeneMANIA, highlighting hub genes (*ITLN1*, *RIPK3*, *CCL19*) and their interactions with chemokines and cell adhesion molecules

To further elucidate the potential molecular mechanisms by which DEGs contribute to OA progression, we performed systematic functional enrichment analyses and constructed a gene interaction network based on the identified DEGs. GO enrichment analysis (Fig. 3A) revealed that the DEGs were significantly enriched in immune and inflammatory biological processes, including leukotriene metabolism, neutrophil chemotaxis, and dendritic cell differentiation. For cellular component terms, the DEGs were primarily localized to the immunoglobulin complex and extracellular region. Regarding molecular function, the DEGs were notably enriched in chemokine receptor binding and G protein–coupled receptor activity. These findings suggest that OA-associated cartilage degeneration is closely related to immune microenvironment dysregulation and extracellular matrix (ECM) metabolic imbalance. KEGG pathway analysis (Fig. 3B) further demonstrated that DEGs were significantly enriched in classical immune and inflammatory signaling pathways, particularly the chemokine signaling pathway (hsa04062) and cytokine–cytokine receptor interaction (hsa04060). Additionally, DEGs were also enriched in pathways related to apoptosis/proliferation (hsa04217) and metabolic dysregulation (hsa01100), implying that impaired energy metabolism may contribute to OA progression through inflammatory cascades.To identify key regulators within the DEG set, we used GeneMANIA to construct a gene–gene interaction network (Fig. 3C). *ITLN1*, *RIPK3*, and *CCL19* emerged as central hub genes, showing strong interactions with inflammatory mediators (e.g., *CXCL12*), adhesion molecules (e.g., *ICAM1*), and necroptosis-related regulators (e.g., *ZBP1*). Notably, *RIPK3* demonstrated extensive interactions with inflammatory cytokines such as *CCL5* and *IL21R*, as well as with other immune-regulatory genes. These findings support the potential role of *RIPK3* as a molecular link between programmed cell death and immune dysregulation in OA.

Collectively, these enrichment and network analyses highlight a coordinated dysregulation of inflammatory signaling, ECM remodeling, and metabolic pathways in OA cartilage. *RIPK3* is positioned as a critical regulator within this network, suggesting its relevance to the underlying pathophysiology of OA.

### Immunofluorescence validation of differentially expressed genes

**Fig. 4:**
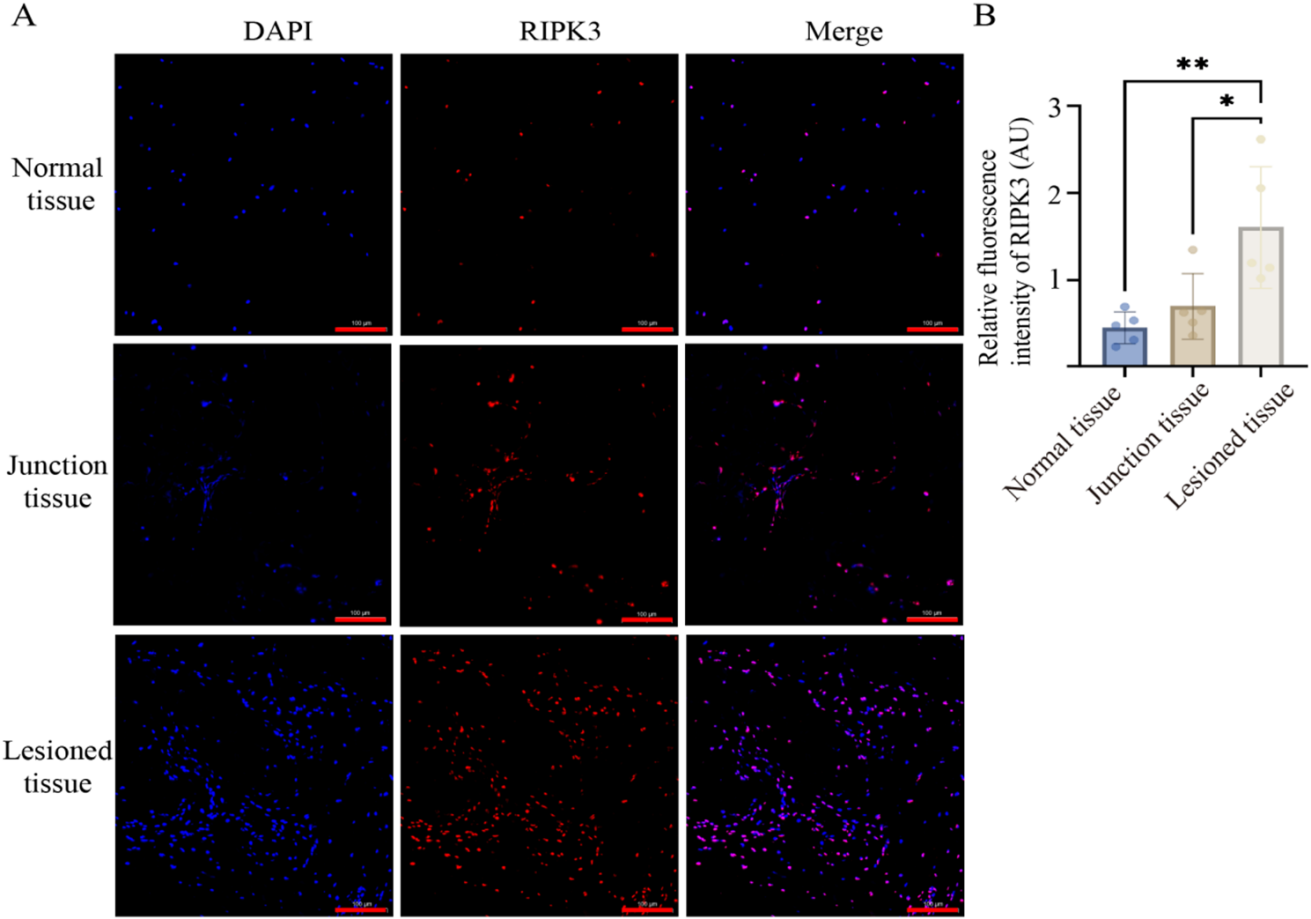
Spatial localization and quantification of RIPK3 expression in osteoarthritic cartilage. **A** Representative immunofluorescence images of RIPK3 expression in the NT, JT, and LT regions (DAPI: blue, RIPK3: red). Scale bars: red-100 µm. **B** Quantification of RIPK3 fluorescence intensity in the NT, JT, and LT regions, presented as box plots. Statistical significance: *p* < 0.01 (**), *p* < 0.05 (*)

Based on transcriptomic findings, immunofluorescence staining was used to validate and localize six candidate proteins (ITLN1, CCL19, XCL1, SIRPG, IL21R, and RIPK3) across anatomically distinct regions of osteoarthritic cartilage. Representative images of RIPK3 expression in NT, JT, and LT regions (Fig. 4A), along with quantitative analysis, showed that RIPK3 fluorescence intensity in LT samples (1.61 ± 0.70 AU) was 3.66-fold higher than in NT samples (0.44 ± 0.19 AU; Kruskal–Wallis test, H = 9.85, *p* = 0.007; Dunn’s post hoc test, LT vs. NT: *p* = 0.005), and also significantly higher than in JT samples (0.69 ± 0.42 AU; *p* = 0.019) (Fig. 4B). For the remaining candidate proteins, fluorescence intensity did not differ significantly among NT, JT, and LT regions (*p* > 0.05). In matched NT and JT samples (n = 5), a consistent directional trend was observed between the transcriptomic fold change (log_2_FC = 2.17, *p* < 0.01) and RIPK3 protein-level upregulation (1.57-fold), supporting concordance between mRNA and protein expression. Notably, RIPK3 protein levels in LT samples were 3.66-fold higher than in NT, exceeding the transcriptomic prediction (linear regression model estimate: 2.14-fold). This discrepancy may reflect post-transcriptional regulation or intrinsic differences between transcriptomic and proteomic profiles in degenerative cartilage tissue.

## Discussion

In this study, we systematically analyzed the degenerative features and key molecular expression patterns of OA cartilage across anatomically distinct regions by integrating transcriptomic profiling, histopathological assessment, and immunofluorescence validation. The results revealed significant regional differences at both the structural and molecular levels, with a particular focus on the sustained upregulation of RIPK3 in degenerated areas, a pattern compatible with local pathological processes during OA progression.

Histological analysis demonstrated progressive cartilage degeneration along the anatomical continuum from NT through JT to LT regions. These changes included thinning of the cartilage layer, tidemark disruption, chondrocyte clustering, and reduced matrix staining, features consistent with the stepwise evolution of OA pathology [16, 17]. This region-specific stratification strategy enabled a more refined understanding of the spatial trajectory of cartilage deterioration.

Transcriptomic analysis identified DEGs between NT and JT regions, many of which were enriched in pathways associated with immune regulation, extracellular matrix (ECM) metabolism, and programmed cell death. GO analysis indicated significant enrichment of inflammatory pathways, such as chemokine signaling (e.g., *CCL5*, *CXCL13*, *CCL19*) and cytokine–cytokine receptor interactions, supporting the notion that cartilage degeneration in OA may be accompanied by synovial inflammation and immune cell infiltration [18, 19]. Previous studies have shown that chemokines like *CCL5* promote inflammatory responses in chondrocytes via NF-κB signaling, further up-regulating matrix-degrading enzymes such as *MMPs* and *ADAMTS*, thereby exacerbating ECM breakdown [20–22]. Our KEGG analysis corroborated this mechanism, showing significant enrichment of *MMP/ADAMTS*-related ECM degradation pathways, which is consistent with the pathological imbalance between cartilage matrix synthesis and degradation in OA [23]. Moreover, the necroptosis signaling pathway, in which RIPK3 plays a key role, was also enriched, consistent with activation of necroptosis-associated programs in degenerative regions [24]. Among the DEGs, RIPK3 stood out: it was consistently upregulated in both JT and LT, concordantly, immunofluorescence showed higher protein abundance in LT, aligning with the transcriptomic trend. Taken together, these observations document spatial enrichment of RIPK3 in degenerative cartilage. While this pattern is compatible with a role in OA pathobiology, our interpretation remains descriptive rather than mechanistic. As a central mediator of necroptosis, RIPK3 has been implicated in inflammatory settings [25–27]. Our study confirmed its upregulation in clinical OA cartilage samples and documented protein-level accumulation in severely degenerated tissue. Additionally, interaction network analysis placed RIPK3 within a network that includes inflammatory mediators (e.g., *CCL5*, *IL21R*) and necroptosis regulators (e.g., *ZBP1*), consistent with a putative interface between immune activation and cell-fate programs. Notably, several *RIPK1* inhibitors are currently under clinical evaluation for the treatment of rheumatoid arthritis [28]. Preclinical reports targeting RIPK pathways in joint disease suggest potential, although further translational research is required to optimize specificity and assess long-term safety [29, 30]. Although necroptosis-linked catabolic programs appear predominant in the degenerative niches we mapped, anabolic signaling is not entirely extinguished; low-dose NGF can promote extracellular-matrix synthesis via TrkA–PI3K/AKT [31].

It is important to note that, apart from *RIPK3*, other candidate DEGs such as *ITLN1*, *CCL19*, and *XCL1* did not show significant regional differences at the protein level by immunofluorescence. This discrepancy may be attributed to spatiotemporal decoupling between mRNA and protein expression, or limitations in translational efficiency, protein localization, degradation dynamics, and antibody specificity [32, 33]. Therefore, single-method protein validation may be insufficient to fully capture dynamic protein expression patterns in complex tissues. Future studies should integrate complementary approaches such as Western blotting, proteomics, or single-cell immune profiling for more robust verification.

This study also has limitations. First, due to tissue availability, the sample size was relatively limited and did not include early-stage OA specimens. Second, although previous studies have implicated RIPK3-mediated necroptosis through MLKL activation [34], MLKL/p-MLKL, NF-κB activity, and RIPK3 phosphorylation were not assayed in this study, and future work will directly test these readouts. Further mechanistic investigations using molecular and functional approaches are needed to clarify *RIPK3*’s precise role in OA pathogenesis. In addition, analyses were confined to cartilage tissue and did not examine the potential role of RIPK3 in other key joint components, such as the synovium and subchondral bone. Therefore, future research should integrate single-cell transcriptomics, multi-omics analyses, and advanced experimental models (e.g., organoids and animal models) to comprehensively investigate the spatial distribution, functional impact, and signaling mechanisms of *RIPK3* in OA progression. These efforts may contribute to the elucidation of OA subtypes and facilitate the development of precision therapeutic strategies—including paracrine, MSC-secretome concepts currently examined in vitro [35].

## Conclusion

This study integrated multi-regional transcriptomic analysis with protein-level validation to characterize spatial molecular differences in osteoarthritic cartilage. *RIPK3* was found to be consistently upregulated in degenerated regions, correlating with histopathological damage, a finding consistent with local cartilage deterioration during OA progression. These findings provide molecular evidence for regional heterogeneity in OA cartilage and offer preliminary support for *RIPK3* as a potential diagnostic marker and therapeutic target. Further studies incorporating functional assays and multi-omics approaches are warranted to clarify its mechanistic role and evaluate its utility in OA stratification and precision therapy.

## Data Availability

The raw RNA-seq datasets generated and analyzed during this study are not publicly available due to data processing constraints but are available from the corresponding author upon reasonable request. Processed data and key differential expression results have been provided as supplementary Excel files.
